# Development of a clinical prediction rule for sepsis in primary care: protocol for the TeSD-IT study

**DOI:** 10.1186/s41512-020-00080-5

**Published:** 2020-08-06

**Authors:** Feike J. Loots, Rogier Hopstaken, Kevin Jenniskens, Geert W. J. Frederix, Alma C. van de Pol, Ann Van den Bruel, Jan Jelrik Oosterheert, Arthur R. H. van Zanten, Marleen Smits, Theo J. M. Verheij

**Affiliations:** 1 Julius Center for Health Sciences and Primary Care, University Medical Centre Utrecht, Utrecht, The Netherlands; 2 Emergency Department, Gelderse Vallei Hospital, Ede, The Netherlands; 3 Star-shl Diagnostic Centres, Etten-Leur, The Netherlands; 4 Department of General Practice, School of Public Health and Primary Care (CAPHRI), Maastricht University Medical Centre (MUMC+), Maastricht, The Netherlands; 5 Department of Internal Medicine, Division Infectious Disease, University Medical Centre Utrecht, Utrecht, The Netherlands; 6 Academic Centre for Primary Care, KU Leuven, Leuven, Belgium; 7 Department of Intensive Care Medicine, Gelderse Vallei Hospital, Ede, The Netherlands; 8 Radboud University Medical Center, Radboud Institute for Health Sciences, Scientific Center for Quality of Healthcare (IQ healthcare), Nijmegen, The Netherlands

**Keywords:** Sepsis; Clinical prediction rule; Diagnosis; Primary care; General practice; Out-of-hours medical care; Practitioner cooperative; Point-of-care testing

## Abstract

**Background:**

Early recognition and treatment of sepsis is crucial to prevent detrimental outcomes. General practitioners (GPs) are often the first healthcare providers to encounter seriously ill patients. The aim of this study is to assess the value of clinical information and additional tests to develop a clinical prediction rule to support early diagnosis and management of sepsis by GPs.

**Methods:**

We will perform a diagnostic study in the setting of out-of-hours home visits in four GP cooperatives in the Netherlands. Acutely ill adult patients suspected of a serious infection will be screened for eligibility by the GP. The following candidate predictors will be prospectively recorded: (1) age, (2) body temperature, (3) systolic blood pressure, (4) heart rate, (5) respiratory rate, (6) peripheral oxygen saturation, (7) mental status, (8) history of rigors, and (9) rate of progression. After clinical assessment by the GP, blood samples will be collected in all patients to measure C-reactive protein, lactate, and procalcitonin. All patients will receive care as usual. The primary outcome is the presence or absence of sepsis within 72 h after inclusion, according to an expert panel. The need for hospital treatment for any indication will be assessed by the expert panel as a secondary outcome. Multivariable logistic regression will be used to design an optimal prediction model first and subsequently derive a simplified clinical prediction rule that enhances feasibility of using the model in daily clinical practice. Bootstrapping will be performed for internal validation of both the optimal model and simplified prediction rule. Performance of both models will be compared to existing clinical prediction rules for sepsis.

**Discussion:**

This study will enable us to develop a clinical prediction rule for the recognition of sepsis in a high-risk primary care setting to aid in the decision which patients have to be immediately referred to a hospital and who can be safely treated at home. As clinical signs and blood samples will be obtained prospectively, near-complete data will be available for analyses. External validation will be needed before implementation in routine care and to determine in which pre-hospital settings care can be improved using the prediction rule.

**Trial registration:**

The study is registered in the Netherlands Trial Registry (registration number NTR7026).

## Background

Sepsis is a life-threatening complication of an infection. Early detection and initiation of adequate treatment is the key factor influencing outcome [1–4]. It is estimated that annually, 49 million people suffer from sepsis worldwide, of which 11 million do not survive [5]. In 2017, the WHO declared sepsis a global healthcare priority and urged member states to improve recognition and treatment of sepsis [6]. Global efforts to reduce mortality and morbidity from sepsis have focused on hospital settings, but patients often present in primary care in the early stages of sepsis. General practitioners (GPs) are confronted with acutely ill patients with a variety of symptoms, signs, and potential diagnoses. Within minutes, they have to decide whether a patient can safely be treated at home or should be referred to a hospital for further assessment.

In the Netherlands, out-of-hours primary care is provided by large GP cooperatives [7]. Patients are only assessed by a GP if the medical complaint cannot wait until the following working day. In contrast to other common time-critical conditions such as stroke and myocardial infarction, patients with sepsis are more likely to contact a GP cooperative instead of an emergency medical service prior to hospital treatment [8]. Data from our preliminary research on patients admitted to an intensive care unit (ICU) due to community-acquired sepsis showed that about half of the patients had prior contact with a GP cooperative. Two thirds of these patients were referred to the hospital after the first contact [9]. The majority of the patients were assessed during a home visit.

In the hospital setting, vital signs are used to screen for sepsis in patient with suspected infections. The systemic inflammatory response syndrome (SIRS) was introduced in 1992 to define sepsis [10]. Besides the white blood count, the SIRS criteria are a heart rate < 90/min, respiratory rate > 20/min, and a body temperature < 36 °C or > 38 °C. As SIRS criteria are often present in patients without serious infections and one in eight patients admitted to the ICU with sepsis were found to lack positive SIRS criteria, a new consensus definition was formulated in 2016. In the “Sepsis-3” definition, the Sequential Organ Failure Assessment (SOFA) score was proposed to diagnose sepsis [1]. As this score is not easy to apply outside the ICU, the quick SOFA (qSOFA) was introduced for rapid bedside assessment. The criteria used in the qSOFA are an altered mental status, systolic blood pressure ≤ 100 mmHg, and a respiratory rate ≥ 22/min. A positive score on two or more parameters predicts an increased risk of mortality. However, the qSOFA is not suitable as a screening tool as it lacks sensitivity [11, 12]. C-reactive protein (CRP), lactate, and procalcitonin (PCT) have all been shown to increase the sensitivity and overall diagnostic performance of the qSOFA [13–15]. To our knowledge, no study has assessed the contribution to the accurate early detection of sepsis in primary care of factors such as symptoms, signs, and biomarkers potentially available as point-of-care tests (POCT) such as CRP, lactate, and PCT.

The aim of the TeSD-IT study (Testing for Sepsis in primary care: Diagnostic and prognostic study Investigating the potential benefits of point of care Testing) is to develop a clinical prediction rule to improve the detection of sepsis while limiting unnecessary referrals in acutely ill patients presenting at the GP cooperative home visits. Clinical signs and symptoms as well as blood tests will be considered as candidate predictors.

## Methods

### Setting and design

We will perform a prospective diagnostic cohort study in the Netherlands in four GP cooperatives (Ede,’s-Hertogenbosch, Uden, and Oss) for out-of-hours primary care. The cooperatives serve a total of approximately 830,000 inhabitants in a mixed urban, suburban, and rural area. The cooperatives are based in or adjacent to regional hospitals.

### Patients

Patients will be recruited during out-of-hours home visits by GPs. Patients only receive home visits when they have acute medical complaints that cannot wait until the next working day and they are not able to visit the GP cooperative location for a clinic consultation. This is decided after telephone assessment by a triage nurse based on the Netherlands Triage System (NTS) [16].

#### Inclusion criteria

1. Acutely ill adult patients (≥ 18 years), receiving a home visit by a GP during OOH
2. Fever, confusion, or general deterioration or otherwise suspected of a serious infection

#### Exclusion criteria

1. Non-infectious cause of the acute complaints (e.g. stroke or myocardial infarction)
2. Hospitalisation within 7 days before the home visit
3. Condition that requires secondary care assessment if there are any signs of systemic infection (e.g. chemotherapy with possible neutropenia)
4. Terminal illness or other reason not to refer the patient to a hospital despite presence of a life-threatening condition.

### Candidate predictors

We selected nine clinical features and three blood tests as candidate predictors for the development of the clinical prediction model (Table 1). Parameters of widely used scoring systems such as the SIRS, qSOFA, and National Early Warning Score (NEWS) [18] were considered, as well as clinical features used in guidelines such as the Netherlands Triage Standard (NTS) and NICE Sepsis guideline [17]. Candidate predictors were selected if there was evidence to suggest that they might usefully contribute to the diagnosis of sepsis and if they can be easily and objectively measured by GPs.

**Table 1:** Candidate predictors eligible for the selection in the prediction model

Type of predictor	Candidate predictor	Measurement method	Measurement unit	Used in
Clinical feature	Age	Inclusion date minus date of birth	Years	NICE guideline [17]
	Body temperature	Tympanic measurement	°C	SIRS [10], NEWS [18], NICE guideline
	Heart rate	IntelliVue MP2/X2	Beats/min	SIRS, NEWS, NICE guideline
	Respiratory rate	IntelliVue MP2/X2 or GP assessment	Breaths/min	SIRS, qSOFA, NICE guideline, NEWS
	Systolic blood pressure	IntelliVue MP2/X2	mmHg	qSOFA, NEWS, NICE guideline
	Peripheral oxygen saturation	IntelliVue MP2/X2	%	NEWS, NICE guideline
	Mental status	GP assessment	Normal/altered	qSOFA, NEWS, NICE guideline
	Rapid progression of illness in last 24 h	GP assessment	Yes/no	NICE guideline
	(History of) rigors in last 24 h	GP assessment	Yes/no	NTS [16]
Blood test	C-reactive protein (CRP)	Siemens, ADVIA Chemistry XPT	mg/l	
	Lactate	StatStrip Xpress	mmol/l	
	Procalcitonin (PCT)	Siemens, ADVIA Centaur XPT	ng/ml	

Candidate blood tests had to be currently used in the hospital setting for the diagnosis and/or prognosis of sepsis, and, preferably, to be available as a point-of-care (POC) test for reasons of implementation. CRP and lactate measurement are part of the standard care in patients with suspected sepsis during assessment in the Emergency Department (ED) in the Netherlands. Procalcitonin (PCT) is not routinely measured in most hospitals, but we decided to include PCT as a candidate predictor as PCT might be superior to CRP [19], and the NICE sepsis guideline recommends research to further evaluate the use of PCT POCT for diagnosing serious bacterial infection and initiating antibiotic therapy. CRP, lactate, and PCT are currently available as POC tests.

### Outcome measures

The primary outcome measure is sepsis within 72 h after inclusion. This will be determined by an expert panel using the Sepsis-3 criteria [1]. The operational definition of sepsis is the presence of infection and a SOFA score (Table 2) of at least two above the baseline (which can be assumed to be zero in patients not known to have preexisting organ dysfunction).

**Table 2:** SOFA score

System	Score			
	1	2	3	4
Respiration				
PaO_2_/FiO_2_, mmHg	< 400	< 300	< 200 with respiratory support	< 100 with respiratory support
Coagulation				
Platelets, × 10^3^/μl	< 150	< 100	< 50	< 20
Liver				
Bilirubin, μmol/l	20–32	33–101	102–204	> 204
Cardiovascular				
Hypotension	MAP < 70 mmHg	Dopamine ≤ 5 or dobutamine (any dose)^a^	Dopamine 5.1–15, or epinephrine ≤ 0.1, or norepinephrine ≤ 0.1	Dopamine > 15, or epinephrine > 0.1, or norepinephrine > 0.1
Central nervous system				
Glasgow Coma Score	13–14	10–12	6–9	< 6
Renal				
Creatinine, μmol/l	110–170	171–299	300–440	> 440
Urine output, ml/day			< 500	< 200

Adapted from Vincent et al. [20]

*PaO*_*2*_ partial pressure of arterial oxygen, *FiO*_*2*_ fraction of inspired oxygen, *MAP* mean arterial pressure

^a^Adrenergic agents administered for at least 1 h (doses given in μg/kg/min)

To limit the workload for the experts, we will appoint three expert panels, each comprising a GP, an emergency physician, and an internist(-intensivist). Each case will be assessed by one panel. All relevant information from medical records from the GP and the hospital when applicable will be presented to the panel (see Additional file 1). If there is no consensus on the primary outcome, the case will be discussed in a face-to-face consensus meeting with all three experts to determine the final outcome. Interobserver agreement between the three panels will be assessed in a selection of 10% of the cases that will be assessed by all panels. Besides the dichotomous primary outcome “sepsis within 72 h”, the likelihood of sepsis will be assigned a numerical score between 0 and 10. This gives information on the remaining uncertainty regarding sepsis classification, providing insight in the degree of bias that may be introduced when calculating diagnostic accuracy measures using dichotomous sepsis classification [21]. Furthermore, the need for hospital treatment is scored between 0 and 10 by the expert panel as a secondary outcome. An average score above 5 will be regarded as a patient that should best be referred to the hospital immediately by the GP and a score ≤ 5 as a patient that does not have to be referred immediately.

Other outcome measures are hospitalisation (length of stay and type of care: ICU or regular ward), maximum SOFA score in the first 72 h, 30-day all-cause mortality, final diagnosis, and medical costs.

### Study procedures

#### Study period

The inclusion period is from June 2018 until April 2020. If in April 2020 the required sample size is not reached, the patients will continue to be recruited until the minimum required number of events has been reached. Follow-up of the patients is 30 days.

#### Procedure during home visit

All patients will receive usual care. Patients will be screened for eligibility during home visits by the attending GP of the GP cooperative. Verbal informed consent will be obtained from the patient or his legal representative. The GP will be (routinely) accompanied by a chauffeur during the home visit. The chauffeurs are used to practically assist the GP during the visit. Portable monitors (Philips intelliVue MP2 or X2) will be available to record peripheral oxygen saturation, automated blood pressure, and heart- and respiratory rate by three lead electrodes on the chest.

The GP will record the assessment of the candidate predictors in a case report form. In addition, the GP will record if he/she has a gut feeling that “something is wrong” and will provide the likelihood of the presence of sepsis at inclusion on a scale from 0 to 10.

All study materials will be taken to the patient’s home in a study bag. The venous samples will be collected by either the GP or an on-call laboratory assistant or nurse within 1 h after inclusion, with a maximum of 8 h. Written informed consent will be obtained prior to the collection of the blood samples. In case the patient is referred to the hospital and the blood samples are not collected by the GP, the study bag will be transported with the patient to the ED. Subsequently, the laboratory assistant on call will visit the patient in the hospital and collect the blood samples. Three blood tubes will be collected: 10 ml for serum, 10 ml for EDTA plasma, and a 2-ml heparin tube. Lactate will be measured immediately afterwards from a single drop of blood taken from the heparin tube, using the StatStrip Xpress (Nova Biomedical) POC test. The remaining blood samples will be taken to the hospital laboratory and divided into six samples of serum and six samples of EDTA plasma. The aliquots will be temporarily stored at the local laboratory at < − 70 °C. Two samples (1 ml serum and 1 ml EDTA plasma) will be transported to the Jeroen Bosch Hospital for CRP and PCT analyses, and the remaining samples (5 × 0.5 ml serum and 5 × 0.5 ml EDTA plasma) will be stored for 15 years at < − 80 °C at the UMC Utrecht for potential future testing.

#### Training and remuneration of personnel

Chauffeurs of the GP cooperatives will be trained in using portable monitors for vital sign measurement and other study procedures. At the GP cooperative in Ede, the chauffeurs will also be trained in the measurement of POC-lactate, as GPs will collect the venous blood samples themselves occasionally. The laboratory assistants and nurses who will be on call for the collection of the blood samples will also be trained in the POC–lactate measurement and other study procedures, including the obtaining of written informed consent. Attending GPs will be informed by an information letter by mail and hard copy at the GP cooperative. Leaflets with a summary of the study procedures will also be available.

#### Follow-up

**Fig. 1:**
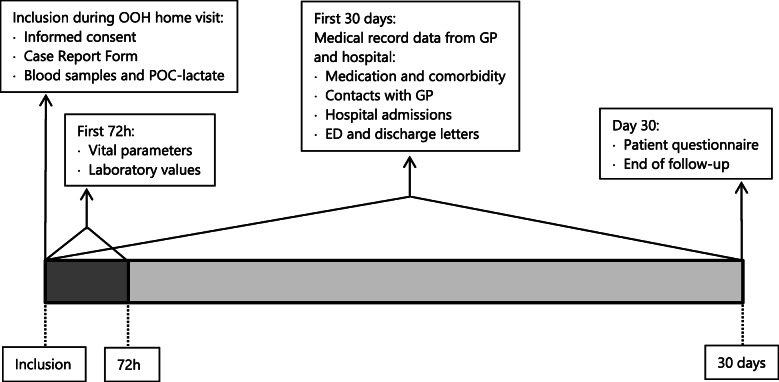
Summary of study procedures

The total follow-up time is 30 days (see Fig. 1). Patients will be asked to complete the EQ5D-5L questionnaire [22] at the end of follow-up to report on their health status: (1) at the day of completion of the questionnaire, (2) before the onset of the recent disease episode (i.e. their health status of at least 1 month ago), and (3) for the worst day they remember from their recent disease episode. Furthermore, patients will be asked to report on consumption of medical resources during the 30-day follow-up period. In case of no response to the questionnaire after 1 week, patients will be contacted once by telephone as a reminder.

#### Data extraction

Relevant medical information will be obtained from the patient’s (regular) GP, the GP cooperative, and the hospital. Information from all hospital admissions in the 30-day follow-up will be retrieved, first by digital search in the local hospital and secondly by manual screening of the GP record for admissions in other hospitals. Medication use and comorbidities before inclusion will be retrieved from GP electronic records, as well as information on any subsequent contacts. The medical record of the assessment at the time of inclusion will be retrieved from the GP cooperative. The following data from the electronic medical record of the hospital will be collected: full reports from ED and hospital discharge; date and time of ED visit, hospital admittance, and discharge (including type of ward); vital signs, EMV score, leucocyte count, thrombocyte count, creatinine, bilirubin, CRP, and lactate measured in the first 72 h after inclusion; cultures taken in the first 72 h after inclusion; radiodiagnostic procedures in the first 7 days after inclusion; antibiotic prescriptions during hospitalisation; and intravenous volume therapy in the first 72 h (defined as more than 1.5 l of fluids in 24 h).

### Sample size

In total, 12 candidate predictors are chosen for the development of the prediction model (Table 1). Using the rule of thumb of 10 events per variable [23], we need 120 patients reaching the primary outcome “sepsis within 72 h after inclusion” in the final dataset. Prior to the start, the prevalence of sepsis based on previous research and literature was estimated to be around 12%. However, preliminary data analysis from the patients included in the study so far indicates the prevalence of sepsis in the study cohort to be around 30–40%. After the first 100 cases will be assessed by the expert panel, we will determine the final target sample size.

### Statistical analyses

#### Descriptive statistics

We will use a combination of IBM SPSS Statistics and R Statistical Software for all analyses. We will start with descriptive analyses on baseline characteristics (age, sex, comorbidities, vital sign measurements and other clinical features, blood tests results, baseline EQ5D-5L score), final diagnosis, hospital admission, ICU admission, length of stay, EQ5D-5L compared to baseline, and 30-day mortality. Results will be stratified based on whether patients do or do not meet the primary outcome sepsis.

#### Data cleaning

Range and distribution of all continuous variables will be graphically inspected, and any outliers (more than three standard deviations from the mean) will be discussed and corrected or removed in case of a data recording error. Any missing data on clinical features or blood tests will be accounted for by applying multiple imputation techniques. Prediction model development and performance will be analysed using the imputed datasets.

#### Development of the prediction model

A multivariable penalized logistic regression model will be developed, based on the variables listed in Table 1, for predicting the primary outcome (sepsis within 72 h after inclusion). We will use a two-stepped approach entering and selecting clinical features first, and blood tests second. In both steps the selection of predictors will be based on a stepwise backward selection, using change in Akaike information criterion (AIC) for selecting the preferred model [24]. The goal is to generate an efficient model by eliminating variables that contribute little to the model’s performance, requiring only measurement of the most important variables in clinical practice.

Continuous predictors in the model will be assessed for linear relationship with the logit of the primary outcome. Transformation of the data and splines will be used if deemed appropriate based on distribution of the data.

The resulting prediction model will be the most accurate prediction model, by making use of continuous measurements of predictors and reflecting non-linear relationships by transformations or splines (optimal model). To make this model workable in daily clinical practice without electronic aids, a second model will be derived (clinical practice model) by categorising or stratifying predictors. Cutoffs for categorisation will be based on a combination of known and commonly used thresholds in clinical practice and optimal thresholds based on the data. This model simplification is likely to induce a performance drop with regard to the full model, which will be assessed during the analysis.

The above procedures will result in the following three models: (1) optimal model with clinical features only, (2) optimal model with clinical features and blood tests, (3) simplified model (with clinical features and blood tests).

#### Performance of the prediction model

The performance of all three models will be determined based on their discrimination and calibration. Discrimination will be evaluated based on the area under the receiver-operator characteristic (AUROC). Calibration will be assessed by plotting observed and expected probabilities and inspecting this plot graphically. Measures of calibration will include calibration slope, calibration in the large, observed/expected (O/E) ratio, and the Brier score [25]. We will perform internal validation for all three models by using a bootstrap simulation. The resulting distribution will reflect optimism and the degree of overfitting [26].

The SIRS criteria, NEWS score, and qSOFA score will be calculated for all individuals in the TeSD-IT study. Diagnostic performance of the existing models will be determined by calculating the same measures of discrimination and calibration as described in the sections above and comparing these with the three models that were developed.

To assess the added value of the prediction models on top of usual care, other outcomes than sepsis will be considered. This is crucial for gaining insight in the net benefit of using the clinical prediction rule in daily practice. For example, when a patient is predicted as non-sepsis by the model, but the patient was referred by the GP, improvement compared to care as usual is only the case if hospital treatment was not needed according to the expert panel. To assess the added value, the proportion of reclassifications within the original contingency tables will be presented for the following outcomes: (1) the gut feeling of the visiting GP that “something is wrong”, (2) the assessment of the visiting GP for the likelihood of sepsis, and (3) the decision of the visiting GP whether or not to refer the patient to the hospital.

#### Cost-effectiveness and budget impact analysis

We will measure costs from a societal perspective, including health care costs and patient costs within and outside the hospital (see Additional file 2 for detailed information). Productivity costs will be ignored as the average age of patients participating will exceed the age of pensioning in the Netherlands. The patient questionnaire as well as follow-up data from hospital and GP medical records will be used for the calculation of total and per-patient costs. The EQ5D-5L scores retrieved from the questionnaire will be used to calculate quality-adjusted life-years (QALYs). Our patient outcome analysis will generate QALYs for different health states that will be used in health economic modelling, such as a complicated sepsis case (including ICU admission), hospital admittance for a suspicion of sepsis, and an infectious disease episode without hospital admission. Different scenarios with different levels of implementation of POCT for sepsis in general practice will be analysed and compared to standard of care: 100% use of the best performing testing strategy, 70%, 30%, and 0% use of POCT for suspicion of sepsis (the latter representing usual care). The budget impact will be assessed using the health economic model that will be built for the economic evaluation, and results will be analysed in a probabilistic way.

## Discussion

The TeSD-IT study is a diagnostic and prognostic study, designed for the development of a clinical prediction rule for the early recognition of sepsis in primary care. A limited number of nine clinical parameters and three blood tests were selected. This will enable us to construct the model using multivariable logistic regression techniques with 120 events included in the dataset. We realise the validity of rule of thumb of 10 event per variable is debated [27]. However, using an alternative sample size calculation suggested by van Smeden [28] results in a similar identical sample size of about 350 patients in case of 12 variables, an outcome rate of 0.35, and rMPSE set at 0.09. We have chosen to recruit patients in the setting of out-of-hours home visits performed by a GP. In this setting, GPs frequently encounter seriously ill patients in whom they instantly have to decide whether or not to refer the patient (immediately) to the hospital. A methodological advantage is that the required sample size is substantially lower than in other primary care settings with a lower incidence of sepsis. However, external validation in other settings and populations will be needed before implementing the clinical prediction rule more broadly.

The diagnosis of sepsis is not straightforward. In 2016, new consensus definitions for sepsis were published, which we try to implement as well as possible. As both the presence of infection as well as organ failure can be equivocal, we will use expert panels to determine the final outcome. The outcome should be clinically relevant for the GP. The rationale of the timeframe of 72 h is that patients who are found septic within this period after GP assessment would likely benefit from immediate hospital referral. Not all patients with organ failure need hospital treatment to recover, and not all patients with severe infections that are treated with intravenous antibiotic therapy have signs of organ failure. However, we believe the diagnosis of sepsis based on the Sepsis-3 definitions is the most relevant endpoint for GPs to differentiate between patients who are likely to benefit from immediate hospital treatment and patients who might be treated at home successfully. The expert panel will also rate the need for hospital treatment for every patient, regardless of the diagnosis. This will enable us to evaluate the effect of the new clinical prediction rule on medically unnecessary referrals.

The expert panels will be instructed to use the SOFA score (increase, due to infection, of ≥ 2 points from baseline) to define the primary outcome “sepsis within 72 h after inclusion”. This is consistent with the Sepsis-3 consensus definition and leads to an objective and reproducible endpoint in the absence of a gold standard. However, this approach introduces the risk of incorporation bias. Parameters included in the SOFA score are more likely to be predictive of sepsis in our model. However, the blood tests (CRP, lactate, and procalcitonin) are not included in the SOFA score, which limits the risk of incorporation bias for these tests. Furthermore, vital signs measured in the first 72 h will be used to calculate the SOFA score and not only at the time of inclusion.

Patients will receive a questionnaire at day 30 measuring EQ5D-5L. The results may be biased due to selective response and poor recall due to sepsis- or age-related cognitive impairment. Imputation of the missing answers on the questionnaires will reduce this form of bias as much as possible. Furthermore, the development of the clinical prediction rule is not affected, as the patient questionnaires will only be used for the cost-effectiveness analyses

To compare the performance of the new clinical prediction rule with usual care, not only the decision to refer the patient to the ED, but also the assessment of the GP of the likelihood of sepsis on a scale from 0 to 10 and the presence of a “gut-feeling something is wrong” will be used. We will examine if the prediction rule will outperform those assessments of the GP in order to likely improve the usual care.

Only three candidate blood tests were selected for the development of the prediction rule. Various other biomarkers have promising diagnostic and/or prognostic properties in patients with suspected sepsis [29, 30]. At the start of this study, sufficient evidence of the additional diagnostic and/or prognostic value above lactate, CRP, and PCT was lacking. However, sufficient blood samples will be stored for retrospective testing of multiple additional biomarkers.

Although to our best knowledge no clinical prediction rules for sepsis in primary care were previously developed, several sepsis screening tools were published for the ambulance setting [31]. However, none of those have adequate inclusion criteria, data collection, and clinically relevant endpoints for use in the primary care setting.

## Supplementary information

**Additional file 1.** Expert panel assessment.

**Additional file 2.** Cost-effectiveness analysis.

## Data Availability

Data will be made available from the corresponding author upon reasonable request after completion of the study.

## Abbreviations

AIC: Akaike information criterion
CRP: C-reactive protein
ED: Emergency department
GP: General practitioner
ICU: Intensive care unit
NEWS: National Early Warning Score
NTS: Netherlands Triage Standard
PCT: Procalcitonin
POC(T): Point-of-care (testing)
AUROC: Area under the receiver-operator characteristic
SIRS: Systemic inflammatory response syndrome
(q)SOFA: (quick) Sequential organ failure assessment score
QALY: Quality-adjusted life year
