# Supplementary material for: Development of a clinical prediction rule for sepsis in primary care: protocol for the TeSD-IT study
Source: Diagn Progn Res. 2020 Aug 6;4:12. doi: 10.1186/s41512-020-00080-5 (PMC7409626; doi:10.1186/s41512-020-00080-5)
Supplement: Supplementary file 1 — Additional file 1. Expert panel assessment. [file 41512_2020_80_MOESM1_ESM.docx]

**Additional file 1**

Expert panel assessment

All members of the expert panel use a secure online tool (Sharepoint) for the assessment of the cases. All relevant medical information from the hospital and GP is presented in this online tool without any personal information of the patients or other persons such as healthcare providers (pseudo-anonymised).

The experts have to answer 5 questions:

1: Is an infection the cause of the acute complaints the patients presented at inclusion? (Yes/No)

2: Is there an pre-existing condition causing a baseline SOFA-score >0?

3: Does the patient meet the criteria for sepsis within 72 hours of inclusion? (Yes/No)

4: What is the likelihood of sepsis on a scale from 0-10?

5: How certain are you of the need for hospital treatment on a scale from 0-10?

The information presented to the experts is the following:

- Letter from the GP visits during with the patient was included in the study.

- Discharge letters form ED visits within 72 hours.

- Discharge letters form hospitalisation starting within 72 hours.

- Medical record from GP contacts in the first 72 hours.

- Microbiology reports from cultures taken in the first 72 hours.

- Radiology reports of all instigations in the first 7days after inclusion.

- Antibiotic treatment during hospitalisation.

- All relevant vital signs and laboratory values of the first 72 hours. The most abnormal value for every calendar day is presented in a table as shown below. The first calendar day starts at the time of inclusion and the fourth calendar day ends exactly 72 hours after inclusion.


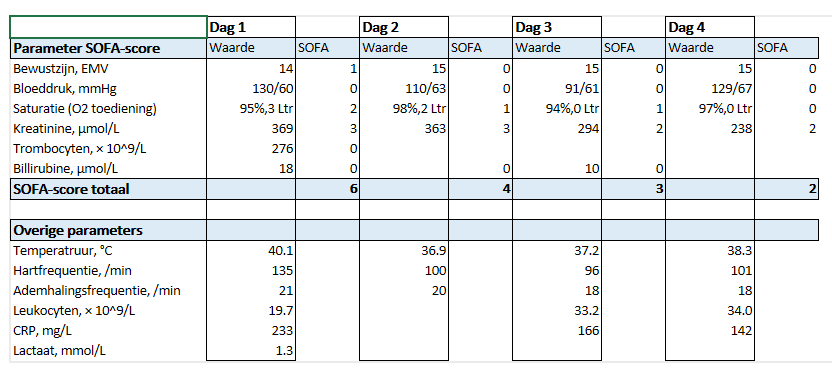


All vital signs and laboratory values that can be digitally extracted from the electronic medical records are used, as well as the vital signs recorded on the case report form at inclusion. Analyses of the blood samples collected for the study are not used to inform the experts.

The experts are instructed to use the Sepsis-3 criteria for the determination of the primary outcome “sepsis within 72 hours”. This means that sepsis is present if an infection is the cause of the complaints in combination with an increase of the SOFA score of ≥ 2 from baseline, within 72 hours. The experts are instructed to take pre-existing conditions into account to estimate what the baseline SOFA score of the patient most likely was.

Handling of missing SOFA points:

Missing SOFA points are only imputed using direct information of the missing parameter. In the absence of direct information, the missing parameters are considered normal.

In case blood results needed for the calculation of SOFA points (platelets, creatinine and/or bilirubin) are not available on one or more of the four calendar days in the first 72 hours, no points are given to that part of the SOFA score. The only exception is in case the missing value is between two days on which the blood values are available. In that case the blood result is imputed with the assumption there was a linear trend between the two measured values. For example, if on Day 1 the creatinine is 130 and on Day 3 100, the imputed value for Day 2 is 115 (Day 4 is not imputed). If the creatinine is 130 on Day 1 and 100 on Day 4, a value of 120 is imputed on Day 2 and 110 on Day 3. Only blood results from the hospital within the first 72 hours are used.

Peripheral oxygen saturation (SpO2) and supplemented oxygen are used to estimate respectively the PaO2 and FiO2 for calculation of the PaO2/FiO2 ratio. Results from blood gas analyses are (outside the ICU) are not taken into account as the amount of oxygen supplied at the time of blood collection is unknown. For the estimation of the PaO2 and FiO2, the following conversion tables are used:

| SpO_2_ (%) | PaO_2_ (mmHg) |  | O_2_ flow (l/min) | FiO_2_ (%) |
| --- | --- | --- | --- | --- |
| ≤80 | 44 |  | 0 | 21 |
| 81 | 45 |  | 1 | 24 |
| 82 | 46 |  | 2 | 28 |
| 83 | 47 |  | 3 | 32 |
| 84 | 49 |  | 4 | 36 |
| 85 | 50 |  | 5 | 40 |
| 86 | 52 |  | ≥6 | 44 |
| 87 | 53 |  |  |  |
| 88 | 55 |  |  |  |
| 89 | 57 |  |  |  |
| 90 | 60 |  |  |  |
| 91 | 62 |  |  |  |
| 92 | 65 |  |  |  |
| 93 | 69 |  |  |  |
| 94 | 73 |  |  |  |
| 95 | 79 |  |  |  |
| 96 | 86 |  |  |  |
| 97 | 96 |  |  |  |
| 98 | 112 |  |  |  |
| 99 | 145 |  |  |  |
| 100 | 200 |  |  |  |

Mean arterial blood pressure (MAP) is calculated using the formula: (2 x diastolic blood pressure + systolic blood pressure)/3.

The AVPU scale (an acronym for "alert, verbal, pain, unresponsive") is used to impute the Glasgow Coma Score. For “A” a score of 15 is imputed, for “V” a score of 12, for “P” a score of 8 and for “U” a Glasgow Coma Score of 3. In case the AVPU scale is extended with “D” for “delirium”, a score of 14 is imputed. In case on the case report form the mental status is recorded as “altered”, a Glasgow Coma Score of 14 is imputed.

In case patients were admitted in the ICU on a specific day within 72 hours of inclusion, SOFA points are obtained from the electronic medical records from the ICU.
