# Supplementary material for: Development of a clinical prediction rule for sepsis in primary care: protocol for the TeSD-IT study
Source: Diagn Progn Res. 2020 Aug 6;4:12. doi: 10.1186/s41512-020-00080-5 (PMC7409626; doi:10.1186/s41512-020-00080-5)
Supplement: Supplementary file 2 — Additional file 2. Cost-effectiveness analysis. [file 41512_2020_80_MOESM2_ESM.docx]

**Additional file 2**

Cost-effectiveness analysis

**Cost analysis**

We propose to measure costs from a societal perspective, including health care costs and patient costs. Productivity costs will be ignored in this study as the average age of patients participating will exceed the age of pensioning in the Netherlands. All costs during follow up will be monitored on an individual basis. Main categories of health care use will be retrieved from both hospital and GP records. In addition, patients will be asked to complete a questionnaire after the follow-up period of 30 days to monitor all health care use that could not be observed from medical records. This mainly relates to professional home care, physical therapy and use of care from relatives. Furthermore, we will ask for type and amount of out-of-pocket payments, e.g. for travel costs and over the counter drugs. We anticipate that most cost categories will have standard unit prices available from the Dutch guidelines on costing research. For the different POCT, we will estimate costs using a bottom up costing approach, including handling of costs of the device (analyzer) as proposed in the Dutch guidelines for costing research. Our cost analysis will generate costs for different health states that will be used in health economic modelling, such as costs of a complicated sepsis case (including ICU admission), costs of hospital admittance for a suspicion of sepsis and costs for an infectious disease episode without hospital admission. Patient outcome analysis Following earlier correct diagnosis of sepsis and the avoidance of unnecessary hospital admissions, quality of life will possibly be better with improved diagnosis of sepsis. However, the measurement of quality of life in patients with severe conditions is hampered by their sometimes devastating health status. Analogous to procedures that were followed in hospitalized pneumonia patients,1 we propose to measure quality of life with a so-called then-test once patients are recovered. At the end of follow-up at 30 days, patients will be asked to complete the EQ5D-5L questionnaire thrice. First, for their current health status at the day of completion of questionnaire. Second, for their health status before onset of the recent disease episode (i.e. their health status of 1 month ago) and third, for the worst day they remember from their recent disease episode. This enables us to calculate QALY losses over this 1 month period. The EQ5D-5L version enables the expression of health status in a single index value for quality of life, necessary for QALY calculations. We will calculate index values according to the algorithm published by Versteegh et al.2 Our patient outcome analysis will generate QALYs for different health states that will be used in health economic modelling, such as a complicated sepsis case (including ICU admission), hospital admittance for a suspicion of sepsis, and an infectious disease episode without hospital admission.

**Budget impact analysis (BIA): general considerations**

The design of the budget impact analysis (BIA) will be a study of different scenarios of either or not introducing POCT for the diagnosis of sepsis in general practice. The BIA will be based on data collected alongside this diagnostic study. It will allow estimation of the financial consequences of introduction of POCT measurements in general practice from the perspective of different stakeholders involved. All cost items needed for the BIA will be derived directly from our diagnostic study, the valuation of those items depends on the perspective taken for the budget impact analysis. The aim of the BIA is to study costs of different scenarios for the nationwide introduction of POCT measurements in general practice to diagnose sepsis in severely ill elderly. We will perform budget impact analyses from two different perspectives. The perspectives to be included in the BIA are:

1. The perspective from the Health care budgetary framework (net-BKZ or government perspective): this will only include direct medical costs reimbursed by basic health insurance coverage. In our study this will include the analysis of the costs of different POCT, of GPs (both regular consultations and out-of-hours consultations), the costs of drugs, of referral to the hospital, the costs of professional homecare and of physical therapy. In the net BKZ perspective only the changes in costs within the basic package of care will be taken into account, including substitution effects when usual care shifts as a result from improved diagnosis of sepsis in elderly. At present, POCT for sepsis are not yet reimbursed for. Positive findings from our diagnostic study, once confirmed in prospective studies, would open a case for reimbursement of POCT diagnostics in this target group, with possible shifts in patient care following from improved diagnosis. Results for the net BKZ perspective will be expressed as potential cost-savings within the health care budget of the ministry of VWS. Results from the net BKZ perspective will be expressed in M€ (millions of Euros).

2. The perspective of health care insurance companies, including all reimbursed health care. Results for the health care insurance companies perspective will be expressed in M€ (millions of Euros). As all sepsis related health care use is most likely also covered in the net BKZ perspective, the cost items to be valued will mainly be similar to those in the net BKZ perspective. However, valuation of costs is different in both perspectives (see below). Different scenarios with different levels of implementation of POCT for sepsis in general practice will be analysed and compared: 100 % use of the best performing testing strategy; 70%, 30% and 0 % use of POCT for suspicion of sepsis (the latter representing usual care). Moreover, the scenario will be analysed that POCT is outsourced by laboratories to GPs, which alters directions and magnitude of costs and reimbursement. Considering that more POC tests are becoming available and popular lately (D-dimer for deep venous thrombosis for example) and that insurance companies will probably require more strict protocols for measurement, such cooperations between laboratories and GPs are becoming more common.

Sensitivity analyses will include variances in costs of POCT and of prevalence of suspicion of sepsis in elderly. The range of the effects to be included in the sensitivity analyses will depend on the results of our proposed trial. As episodes of sepsis usually do not exceed a 30-day period, the time horizon for the BIA will be four years. BIA results will be reported separately for each year within the time horizon and indexation will be applied. The budget impact will be assessed using the health economic model that will be built for the economic evaluation and results will be analysed in a probabilistic way.

**Budget impact analysis: cost analysis**

Different perspectives for the BIA merit different valuation of resource use. The total number of patients eligible for the intervention will be estimated based on data collected in this diagnostic study and extrapolation to national level. Resource utilization is calculated by multiplying the number of eligible patients with the resource utilization patterns obtained from our data collection. Different prices will be used to value resource use depending on the perspective of the analysis: actual NZA tariffs for the government perspective, and average tariffs NZA for the insurer perspective. Both resource use and annual costs will be presented over a 4 year period for all perspectives. Different implementation scenarios will be evaluated (implementation rate will be varied between 0% and 100%). Aggregated and disaggregated (e.g. GP care, secondary care) total costs per year will be presented for the different perspectives and scenarios.

**References**

1. Mangen MJJ, Huijts SM, Bonten MJM, de Wit GA. The impact of community-acquired pneumonia on the health-related quality of life of elderly. BMC Infect Dis 2017;17(1):208. doi: 10.1186/s12879-017-2302-3

2. M Versteegh M, M Vermeulen K, M A A Evers S, et al. Dutch Tariff for the Five-Level Version of EQ-5D. Value Health. 2016 Jun;19(4):343-52. doi: 10.1016/j.jval.2016.01.003. Epub 2016 Mar 30. PubMed PMID:27325326.
